# DIAMUND: Direct Comparison of Genomes to Detect Mutations

**DOI:** 10.1002/humu.22503

**Published:** 2013-12-25

**Authors:** Steven L Salzberg, Mihaela Pertea, Jill A Fahrner, Nara Sobreira

**Affiliations:** 1 Center for Computational Biology, Johns Hopkins School of MedicineBaltimore, Maryland, 21205; 2 McKusick-Nathans Institute of Genetic Medicine, Johns Hopkins School of MedicineBaltimore, Maryland, 21205

**Keywords:** variant detection, computational biology, bioinformatics, exome sequencing, sequence alignment

## Abstract

DNA sequencing has become a powerful method to discover the genetic basis of disease. Standard, widely used protocols for analysis usually begin by comparing each individual to the human reference genome. When applied to a set of related individuals, this approach reveals millions of differences, most of which are shared among the individuals and unrelated to the disease being investigated. We have developed a novel algorithm for variant detection, one that compares DNA sequences directly to one another, without aligning them to the reference genome. When used to find de novo mutations in exome sequences from family trios, or to compare normal and diseased samples from the same individual, the new method, direct alignment for mutation discovery (DIAMUND), produces a dramatically smaller list of candidate mutations than previous methods, without losing sensitivity to detect the true cause of a genetic disease. We demonstrate our results on several example cases, including two family trios in which it correctly found the disease-causing variant while excluding thousands of harmless variants that standard methods had identified.

## Introduction

The use of genome sequencing to discover mutations responsible for disease, including both inherited diseases and cancer, has exploded in recent years. Both whole-genome and whole-exome sequencing have been employed in thousands of individuals in an effort to detect genetic changes that might be responsible for disease phenotypes. Many successes have followed the 2009 reports that used exome sequencing to identify mutations for Miller Syndrome [Ng et al., 2010] (which was found after sequencing the exomes of just four individuals), Bartter syndrome [Choi et al., 2009], and Freeman–Sheldon syndrome [Ng et al., 2009].

The high-quality, nearly complete human reference genome [The International Human Genome Sequencing Consortium, 2001], currently GRC37/hg19, serves a critical role in all of these studies. Standard protocols for analysis begin by aligning all the sequences from proband and family members to GRC37 and then using computational methods to identify high-confidence differences, including single-nucleotide polymorphisms (SNPs), small insertions and deletions (indels), and copy-number variants. However, because of natural variation in the human population, comparing a random individual to GRC37 yields on the order of 50,000–100,000 variants within the exome alone. (Exome sequencing typically captures 50–60 million base pairs, about 2% of the genome.) Sequencing of the entire genome yields millions of variants; for example, Roach et al. (2010) found 4.5 million SNPs when they compared a family quartet to the reference genome. Finding the variant that causes a Mendelian disease, which is often a single mutation among this huge set of candidates, becomes analogous to looking for a needle in a haystack. Recent work has focused on the development of statistical models to reduce these large sets of candidate mutations (e.g., Strelka [Saunders et al., 2012] and FamSeq [Peng et al., 2013]), but the essential problem remains: any method that begins by alignment to the reference genome will initially identify a very large number of naturally occurring sequence variants.

Exome analysis algorithms employ a series of filters to narrow down the set of 50,000 or more variants to a manageable set, which can then be evaluated individually and validated with follow-up experiments. These filters are ad hoc criteria that sometimes filter out the true variant of interest. For example, computational methods sometimes filter out all variants that have been observed in large SNP databases such as dbSNP, or alternatively variants observed at a frequency >1% in a large project such as the 1000 Genomes Project. This can be highly effective as a filter, removing thousands of harmless SNPs that represent common population variants. However, this step implicitly assumes that none of the SNPs in these databases are disease-causing. This assumption may be false, even when SNPs are collected only from healthy individuals, due to incomplete penetrance, unrecognized disease in the subjects, and other factors.

Analysis pipelines usually eliminate noncoding variants as well, which removes large numbers of variants but at the same time discards mutations in splice sites and regulatory sites that might be functionally significant. Although these and other filters might unintentionally eliminate the true cause of disease, without them the number of candidate mutations found in an exome sequence may be overwhelming. The problem is even worse for whole-genome sequencing, where the number of true but clinically irrelevant variants will be 50 times greater.

Here, we introduce a new method, Diamund (direct alignment for mutation discovery), which takes a different approach to exome and whole-genome analysis, and as a result produces dramatically smaller sets of candidate mutations. Rather than aligning all samples to the reference genome, we align the sequences directly to one another. This method is designed primarily for two types of analyses: (1) self-comparisons, where diseased tissue is compared with normal tissue from the same individual, and (2) family studies, where the differences among the DNA sequences from the subjects are far fewer than the differences between any subject and the reference genome.

Our method does not require that the raw sequencing reads, usually numbering 100 million or more for a whole exome, be aligned to the GRC37 reference genome, nor does it require a complex genome assembly or an all-versus-all alignment of these large data sets. As we explain in detail below, we use a more efficient algorithm that allows us to quickly find sequences that are unique to any sample.

We have implemented and tested Diamund on exomes representing two types of analysis problem. First, we considered self-comparisons, in which DNA from primary cultured fibroblasts derived from diseased tissue in an affected individual was compared with DNA from nondiseased primary cultured fibroblasts from the same individual. For the analysis of tumor cells or other somatic mosaic genetic abnormalities, this direct comparison should yield a smaller set of variants than an analysis that first compares all sequences to the reference genome. Second, we looked at three parent–child trios in which a de novo mutation in the child was suspected to be causing disease. The standard algorithm would compare all three individuals to the reference genome, generating very large lists of variants, many of which are shared by the child and a parent. By comparing the child's DNA directly to both parents, we can quickly identify all de novo mutations, without losing sensitivity and without detecting family-specific variants that add noise to the process. For each of these problems, the number of true de novo mutations is very small, obviating the need for the aggressive filters that exome and whole-genome pipelines use, which might eliminate the true variant of interest.

De novo mutations may account for a high proportion of Mendelian disorders. Yang et al. recently reported [Yang et al., 2013] on exome sequencing of 250 probands and their families, among which they identified 33 patients with autosomal dominant and nine with X-linked diseases. Of these, 83% of the autosomal dominant and 40% of the X-linked mutations occurred de novo.

In addition to generating fewer false positives, direct comparison between samples within a family, or between affected and unaffected tissue, allows for detection of mutations in regions that are entirely missing from the reference genome. It has already been shown that some human populations have large shared genomic regions, often spanning many megabases [Li et al., 2010], which are missing entirely from the human reference genome. These include novel segmental duplications [Schuster et al., 2010] as well as entirely novel sequences. If a mutation of interest happens to fall in one of these regions, then conventional methods will be guaranteed to miss it. Our direct comparison algorithm, in contrast, includes these regions and is quite capable of finding mutations within them.

An important caveat is that Diamund is not intended to solve the more general problem of variant detection in any sample. It is designed to take advantage of very closely related samples where direct between-sample comparisons can more effectively identify mutations present in just one or a subset of the samples.

## Methods

Diamund begins with two or more sets of DNA sequences, or “reads,” generated by a sequencing instrument. Here, we describe the algorithm as applied to three trios consisting of an affected individual (or proband) and two unaffected parents. Specializing the algorithm to two samples, where one is normal and the other is diseased (e.g., cancerous) tissue from the same individual, is straightforward.

**Figure 1:**
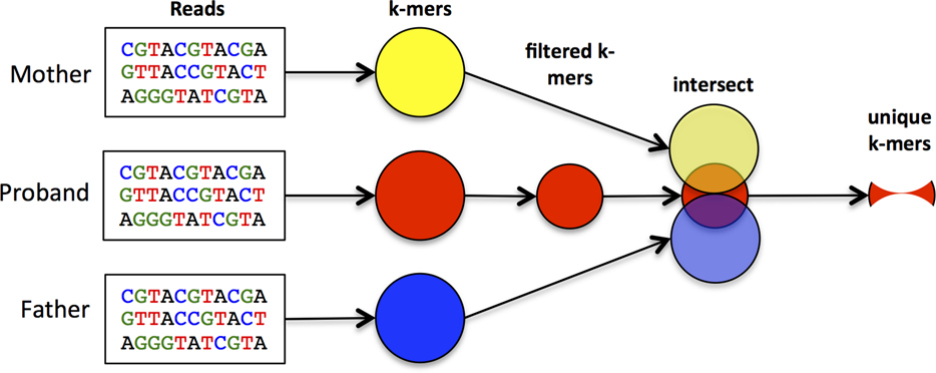
Outline of initial steps in the Diamund algorithm, which identifies all *k*-mers unique to an affected proband and missing from both unaffected parents. The first step identifies *k*-mers, after which the proband data are filtered to remove *k*-mers resulting from sequencing errors. Intersecting all three sets identifies *k*-mers that are unique to the proband.

One way of directly comparing two or more genomes is to assemble each data set de novo, using any of several next-generation sequence assemblers [Schatz et al., 2010], and then compare the assemblies using a whole-genome alignment algorithm such as MUMmer [Delcher et al., 1999; Kurtz et al., 2004]. However, whole-genome assembly is computationally costly and can produce erroneous assemblies, which in turn might create even larger problems than aligning all reads to the reference genome. Instead, Diamund uses a direct approach in which we count all sequences of length *k* in all the reads, for some fixed value of *k*, and then compare these *k*-mers to one another. Here, we outline the 10 major steps of the algorithm; the initial steps are illustrated in Figure1.

Step 1: We utilize an efficient parallel algorithm, Jellyfish [Marcais and Kingsford, 2011], for the *k*-mer counting step. This first step converts the reads for each exome (or genome) to a set of *k*-mers, which should in theory be a much smaller data set: the number of *k*-mers in an exome is equivalent to the length of the exome, 50–60 Mbp using current exome capture kits. However, the initial set is dramatically larger, due primarily to sequencing errors, which we address below. We sort each set of *k*-mers to allow for efficient intersection operations in subsequent steps. Sorting *N k*-mers requires *O*(*N* log* N*) time, after which computing the intersection with another set of *k*-mers requires only *O*(*N*) time.
Step 2: The second step in the Diamund algorithm removes all *k*-mers from the proband (but not from the unaffected samples) that are likely to represent sequencing errors. Note that every sequencing error introduces *k* new *k*-mers. If *k* is sufficiently large, then virtually all of these *k*-mers will be unique, i.e., they will not occur in the genome or elsewhere in the reads. Combined with the fact that exome coverage is usually very deep, we can safely assume that any *k*-mer that occurs just once represents an error.

After empirical observations of multiple exomes, we observed that even *k*-mers occurring more than once are usually errors. Due to biases in sequencing technology, exome data sets may contain erroneous *k*-mers that occur 10 or more times, particularly for regions that contain very deep coverage (which can exceed 1000-fold for some exonic targets). For the exomes we have analyzed, average coverage is approximately 80–100×, which means that a novel, heterozygous mutation should have 40–50× coverage. Even in regions with lower coverage, novel mutations should have 20 or more reads (and *k*-mers) covering them. Note that in the case of mosaicism, a much lower proportion than 50% of the reads might contain the mutation; the software can be adjusted to report such cases.

Given these observations, at this stage, we discard all *k*-mers that occur fewer than 10 times. We tested different values before choosing 10 as the default value, and this can easily be adjusted for data sets with lower or higher coverage. In our tests, a minimum value of 10 excluded an extremely small number of true *k*-mers.

Step 3: After removing likely sequencing errors, some *k*-mers may remain due to vector contamination. We precompute all *k*-mers in known vectors, taken from the UniVec database (www.ncbi.nlm.nih.gov/tools/vecscreen/univec), and remove these from the exome representing the proband (or the diseased tissue, in the case of normal vs. diseased tissue comparisons).

We also observe that any *k*-mer that occurs in the reference genome is probably not the cause of disease. We precompute all *k*-mers from the targeted regions of the GRC37 genome, and remove these “normal” *k*-mers from the proband's data. Note that this set can easily be expanded to include a larger set of variants known to be harmless.

Step 4: After computing all *k*-mers in the reads from the proband and both parents, the third step computes the intersection between proband and mother, and separately between proband and father (Fig.1). We collect all *k*-mers unique to the proband but missing from the mother, and repeat this step for the father. We then intersect the two resulting files to give us a single file that contains all *k*-mers found in the proband but missing from both unaffected parents. These form our initial set that should contain any de novo mutations in the affected individual.
Step 5: At this point, Diamund usually has reduced the initial set of *k*-mers over 10,000-fold, leaving between 2,000 and 65,000 *k*-mers (Table1). For the fifth step, we collect the reads containing these *k*-mers. This requires us to align the *k*-mers back to the original reads, because the Jellyfish *k*-mer counter does not keep track of the source of each *k*-mer. Diamund can use either of two efficient alignment systems for this step: MUMmer [Delcher et al., 1999; Kurtz et al., 2004], a suffix tree-based algorithm that rapidly finds exact matches; or Kraken [Wood and Salzberg, 2013], a fast sequence classifier that we modified to provide the output needed by our system. Kraken is the default choice because it is significantly faster. In our experiments, the number of reads identified in this step ranged from 4,400 to 148,000 (Table1).
Step 6: Despite every effort to screen reads for contamination, some small fragments of vector sequences often still remain in the reads. If these vectors happen to contaminate only the proband (or affected) data set, they will appear to be novel mutations. We eliminate these by comparing the reads identified in the previous step to the UniVec database (www.ncbi.nlm.nih.gov/tools/vecscreen/univec) using the vecscreen program, and removing any reads with vector sequence. Note that running vecscreen on the original data would be extremely demanding computationally, but because the number of reads at this step has been reduced approximately 1,000-fold, it is relatively fast.
Step 7: At this point, the read data are small enough to assemble into small contigs (contiguous sequences), which should contain the mutations of interest. Diamund uses the Minimo assembler [Treangen et al., 2011] to assemble the reads in this step. This step typically produces from a few hundred to several thousand contigs. All the contigs are very short because, by construction, every read used for a given contig must contain the mutation. Thus, for 100-bp reads, the contigs can be no longer than 199 bp.
Step 8: Due to slight differences between reads caused by sequencing errors, the assembly step often produces contigs that overlap one another but which were not merged together. To address this redundancy, we next align all the contigs as well as any unassembled reads to the reference genome using Bowtie2 [Langmead and Salzberg, 2012]. If multiple contigs or reads map to the same location, we collapse them into a single contig. We then retain all contigs containing at least four reads. Note that this is the first step in the Diamund algorithm that uses the reference genome, and that we do not use the reference to identify mutations, but only to aid in merging the contigs.
Step 9: Another reason that a sequence may appear to contain a de novo mutation is that the exome sequencing failed to capture a region in one of the parents, even though the region was targeted. For example, if a child has a heterozygous site but only one parent's DNA was captured by the exome sequencing protocol, that site will appear to contain a sequence not found in either parent. To eliminate these false positives, we next align the reads from both parents to the contigs assembled in the previous step.

**Table 1:** Illustration of the Data Reduction at Each Step from Raw Reads to a Final Set of Mutated Loci

	Data remaining at the end of step			
Filtering step	Disease/normal pair	Family trio BH1019	Family trio BH2041	Family trio BH2688
Number of reads from proband/diseased tissue	118,414,556	84,201,820	75,877,750	103,527,644
Number of 27-mers in proband/diseased tissue	911,738,627	795,477,167	517,272,851	1,088,610,020
Number of *k*-mers with count >10	77,903,885	61,805,320	64,719,150	113,066,951
Remove vector sequence	77,898,848	61,800,798	64,713,995	113,062,417
Eliminate *k*-mers found in reference GRC37 exome	17,821,359	9,385,347	10,730,208	50,535,681
Eliminate *k*-mers found in parent exomes/normal tissue	10,568	65,352	20,130	2,006
Identify reads containing *k*-mers	32,829 reads	148,496	46,454	4,404
Remove reads containing vector	15,260	125,648	38,799	2,760
Number of contigs after assembly	2,147	13,189	3,755	359
Number of contigs with >3 reads after merging contigs	279 contigs	1,437	701	71
Identify variants covered by reads from normal tissue	55 contigs	5	6	2
Keep variants with >5% coverage	42 variants	5	6	2
Find variants in coding regions	14 variants	3	3	1
Remove synonymous SNPs	10 variants	2	3	1

For this step, we use Bowtie2 to align all reads from each parent to the contigs. For each contig that was mapped to the reference genome, we extend it by the length of a read (e.g., 100 bp) on either end, so that we can capture reads that align only partially to the contig. If the contig does not map to the genome (i.e., it represents a novel insertion), we use it but do not extend it. After aligning reads from the parents to these contigs, we align them to the reference genome to determine whether they have a better alignment to another location. In those cases, we discard the reads.

We then scan the alignments to determine whether the putative de novo mutations within the contigs are covered by reads from both parents. Contigs that are not covered are removed from further consideration. Any contigs that fail to map to the reference genome are reported separately; these represent mutations in regions not found in the reference, and require manual analysis.

Step 10: Finally, using the alignments to the reference genome, we compute precisely where the de novo mutations occur. We call variants using the mpileup function in the Samtools package [Li et al., 2009]. We then analyze these locations to determine whether they fall in protein coding regions and whether they cause amino acid changes.

## Results

To demonstrate Diamund in practice, we describe its step-by-step results on several sets of exomes from anonymous subjects (Table1), all sequenced as part of a large-scale study at the Baylor–Hopkins Center for Mendelian Genomics, part of an international research effort (e.g., Hanchard et al. [2013]) to determine the genetic causes of Mendelian disorders. In one experiment, exome sequences were generated from cultured dermal fibroblasts derived from normal tissue and from diseased tissue from the same individual. In three other experiments, exomes were sequenced from trios comprising an affected proband and two unaffected parents, and the exomes were compared to identify de novo mutations found only in the proband.

Table1 shows how each major step of the algorithm reduces the initial set of reads to a smaller set of reads, *k*-mers, or contigs containing candidate mutations. Beginning with the reads, we computed all *k*-mers of length 27 (a value that can easily be changed) in the proband and both parents (for the trios) or in the diseased and normal patient-derived fibroblasts.

In every case, as shown in Table1, the number of unique *k*-mers is initially enormous, over 1 billion for some exome samples. Because the exome regions targeted in these experiments contain only 65 million *k*-mers, most of these *k*-mers must be the result of sequencing errors. The first major filter reduces this number by a factor of more than 10, leaving 62–113 million *k*-mers. Removing *k*-mers found in the exome of the reference genome reduces this set even more, leaving 9–51 million *k*-mers.

The most dramatic reduction comes when we remove all *k*-mers that are found in either of the parental exomes (for normal vs. diseased samples, we remove *k*-mers found in the normal tissue). This removes all variants common to the family, including many that would initially show up as SNPs in an alignment to the reference genome. In the four experiments shown in Table1, the number of *k*-mers left after this step ranged from 2,006 to 65,352. These represent a reduction in the number of candidates by a factor ranging from 140-fold to 25,000-fold. In all cases, the number is small enough to quickly assemble the reads containing those *k*-mers into a set of small contigs.

Assembly of the reads generated between 359 and 13,189 contigs. After merging contigs further and eliminating those supported by only two to three reads, these sets were reduced to 71–1,437 contigs.

At this stage, many of the variants may still be artifacts caused by lack of coverage in one of the parental exomes (see Step 7 above). Diamund only retains contigs if the candidate mutations within them are covered by both parents (or in the case of normal versus disease pairs, covered by the normal exome). This step uniformly reduced the list of variants to a set that was small enough to review manually, ranging from two to 10 variants for the family trios, and 55 for the disease-normal exome pair.

At the very end of the pipeline, we align the contigs to the reference genome to identify variants that occur within protein coding regions, and to determine which cause changes in the amino acid sequence. For the family trios, this step left us only one to two variants in each case. The disease-normal pair yielded 10 variants with nonsynonymous changes. Note that contigs that fail to align to the reference are still reported by the algorithm, and the variants within them can be explored for variants of functional significance. By definition, though, these contigs will not have close similarity to known protein coding regions and they will have to be analyzed using ad hoc methods.

The exomes shown in Table1 were also analyzed using the variant detection system GATK, including its base quality score recalibration, indel realignment, and variant discovery methods [DePristo et al., 2011]. The numbers of variants reported by GATK are compared with Diamund in Table2. (Note that GATK is a general purpose variant detection method with many capabilities beyond family trio analysis, and that Diamund does not replace these other capabilities.) For this comparison, we used Diamund's results prior to the final steps in which it looks at coding versus noncoding variants, which corresponds more closely to the output of GATK.

**Table 2:** Comparison of the Number of De Novo Mutations Found by DIAMUND and GATK when Comparing Exomes from Family Trios and Exomes from Diseased and Normal Cultured Fibroblasts from the Same Individual

Method	Disease/normal pair	Family BH1019	Family BH2041	Family BH2688
Diamund	42	5	6	2
GATK: variants found in affected individual (or diseased tissue)	62,962	60,173	67,034	85,226
GATK: variants in affected individual/diseased tissue but not in unaffected	1,644	7,726	5,621	953

The initial comparison of a proband's exome to the reference genome produced between 60,000 and 85,000 variants for these samples. After filtering to remove variants found in either parent (or the healthy tissue), these sets were reduced substantially, leaving between 953 and 7,726 variants identified as unique to the affected individual. By comparison, Diamund found two to 42 variants. Using the Atlas-SNP variant detection algorithm [Shen et al., 2010], a recent exome sequencing study of 250 probands [Yang et al., 2013] reported finding approximately 400 to 700 variants per sample. This illustrates that even with exomes from both parents available, an alignment protocol that first aligns reads to the reference genome yields a far larger set of candidate mutations than Diamund's direct alignment algorithm.

## Computational Speed

Diamund has been designed to use the most efficient algorithms available for each step in its algorithm. These include Jellyfish [Marcais and Kingsford, 2011] for *k*-mer counting, Bowtie2 [Langmead and Salzberg, 2012] for alignment, and Kraken [Wood and Salzberg, 2013] for mapping *k*-mers back to reads. For the three family trios analyzed here, running the entire pipeline takes 5–7.5 hr per trio using eight threads for those steps that permit parallel execution. (Software was run on a 48-core Dell PowerEdge R815 with 2.1 GHz AMD Opteron processors and 256 GB of RAM.) By comparison, on the same hardware, GATK takes approximately 24 hr for each exome, using a similar number of parallel threads, plus approximately 2–3 hr more, depending on the number of reads, to align the raw reads to the genome. Thus, the total time required by Diamund is far less than GATK, and is approximately the same as that required solely by the initial alignment step: aligning three exomes to the GRC37 reference genome takes approximately 6 hr, using the fastest available software and 10 parallel threads.

## Discussion

Because Diamund reports very few candidate mutations, all of them can realistically be investigated as possible disease-causing mutations. These include noncoding variants, some of which might affect splicing or transcription, but which are usually ignored in an effort to pare down the list of candidates. It also allows investigators to focus their validation efforts on a small set of likely candidate mutations, in contrast to methods that yield many more candidates.

For example, in family trio BH1019 (Table1), Diamund found just five de novo mutations in the proband, two of which were nonsynonymous. One of these caused an amino acid change, and the other introduced a premature stop codon (c.716C>G [p.Ser239Ter]) in the *NFIX* gene (MIM #164005, NM_002501.2). Notably, *NFIX* haploinsufficiency has been shown to cause Sotos syndrome 2 [Malan et al., 2010; Yoneda et al., 2012]. The *NFIX* gene had not been interrogated prior to this study, although the proband had previously had negative *NSD1* (MIM #606681) sequencing to rule out Sotos syndrome 1 [Tatton-Brown et al., 2005]. Based on this analysis, Sotos syndrome-2 (MIM #614753) is the most likely diagnosis for this individual.

In family trio BH2041 (Tabel 1), Diamund found six de novo mutations, three nonsynonymous. The proband had a phenotype similar to Say–Barber–Biesecker–Young–Simpson syndrome (MIM #603736), which is sometimes associated with mutations in *KAT6B* (MIM #605880, NM_01166419.1), but no mutations in that gene were identified. Nevertheless, one of the three de novo nonsynonymous mutations (c.356C>T [p.Thr119Met]) identified by Diamund was in *HDAC8* (MIM #300269), one of the genes known to cause Cornelia de Lange syndrome 5 (MIM #300882). Reevaluation of the proband's phenotype suggested that Cornelia de Lange syndrome is the most likely diagnosis for this individual.

In family trio BH2688, Diamund identified one de novo mutation (c.1192C>T [p.His398Tyr]) in *TMTC4* (NM_032813.2) in the proband, who carries a possible diagnosis of congenital sulprabulbar paresis (MIM #185480). Although *TMTC4* has not previously been associated with any genetic disorder, we are now evaluating it as the possible cause of this condition. The three variants described here have been submitted to the Clinvar database, http://www.ncbi.nih.gov/clinvar.

Finally, we applied the analysis to DNA isolated from primary cultured dermal fibroblasts derived from diseased versus normal tissue from the same individual affected with a novel connective tissue disorder, which is presumed to involve somatic mosaicism. Diamund identified just 42 variants present exclusively in the affected tissue, compared with 1,644 variants previously identified using a more traditional analysis of the affected cells compared with the normal cells (Table2). After removing synonymous variants and those outside of protein coding regions, Diamund reported 10 variants, whereas our traditional analysis yielded 177, although this latter number was reduced threefold by removing commonly seen variants. Only five of the 10 variants identified by Diamund were also identified by our standard analysis, though many additional false positive variants were also identified by the latter. Four of the five variants identified by Diamund were validated by Sanger sequencing as being present in the diseased but not in the normal fibroblast DNA. In other words, of the six true positive variants present exclusively in the diseased cells, two were identified by both approaches, two were identified by Diamund only, and two were identified by traditional analysis only. Notably, four out of seven (57%) of the 10 total variants identified using Diamund were validated by Sanger sequencing and thus were confirmed as being present in diseased cells but not in the normal cells. This was a much better validation rate, as compared with just four of 28 (14%) of variants identified using a more traditional method, suggesting that Diamund has a higher specificity when compared with our traditional analysis, which is based on initial alignments to the reference genome. An important caveat is that due to the possible presence of mosaicism, the failure to validate by Sanger sequencing does not always imply that the variant in question was a false positive.

As these results demonstrate, direct alignment of DNA sequences from related individuals is faster and more sensitive, with fewer false positives, than the standard approach of aligning everything to the reference genome. Our method automatically excludes common variants in an individual or a family, allowing researchers to focus on a much smaller set of variants that are truly novel within the tissue or sample under investigation. The speed advantage of the Diamund algorithm will be even more substantial when applied in a whole-genome context.

One limitation of Diamund is that, as currently implemented, it can only find de novo mutations, that is, mutations that are present in one sample and missing in another. This has immediate applicability in studies of cancer versus normal tissue, and in analyses of family trios. However, the algorithm can in principal be extended to detect other types of mutations, such as compound heterozygotes, in which a child inherits different abnormal alleles in the maternally and paternally transmitted copies of the same gene, one from each parent, and future development will address this and related questions.

Software availability: The Diamund software is open source and freely available at http://ccb.jhu.edu/software/diamund.
